# Deficiency of NF‐Erythroid 2‐Related Factor 2 Exacerbates the Scar Through ERK Signaling Pathway

**DOI:** 10.1002/iid3.70361

**Published:** 2026-02-23

**Authors:** Xueyuan Hao, Mengke Sun, Ruotong Wang, Hong Chen, Junkai Huang, Jing Luo, Haoyue Yu, Yingxi Li, Weibin Xing, Xiaoxia Li, Lizhi Hu

**Affiliations:** ^1^ Immunology Department, Key Laboratory of Immune Microenvironment and Disease (Ministry of Education) Tianjin Medical University Tianjin China; ^2^ Department of Dermatology Tianjin Academy of Traditional Chinese Medicine Affiliated Hospital Tianjin Hongqiao District China; ^3^ College of Basic Medical Sciences Tianjin Medical University Tianjin China; ^4^ Department of Ophthalmology Second Hospital of Tianjin Medical University Tianjin China

**Keywords:** collagen, ERK signaling pathway, fibroblasts, Nrf2, scar formation

## Abstract

**Background:**

Scar, primarily composed of poorly structured and disordered collagen, is mainly characterized by excessive fibroblast activation and abnormal collagen deposition. Previous studies have shown that fibroblast activation is closely related to the imbalance of inflammatory responses and reactive oxygen species. NF‐erythroid 2–related factor 2 (Nrf2) is an important transcription factor involved in regulating inflammation and oxidative stress. Although current evidence suggests that Nrf2 influences the occurrence of skin fibrosis, the role of Nrf2 in scar formation remains unknown.

**Methods:**

In this study, we used qRT‐PCR, Western Blot, and Immunohistochemical/Immunofluorescent staining to explore the role of Nrf2 in wound healing and scar formation.

**Results:**

The deficiency of Nrf2 delayed the process of wound healing and exacerbated collagen deposition. Moreover, significant elevations in the mRNA expression of collagen synthesis‐related genes were observed in Nrf2‐deficient mice in vivo and in vitro. Meanwhile, Nrf2 deficiency also exacerbated inflammatory responses, leading to abnormal fibroblast activation and sustained activation of the ERK signaling pathway.

**Conclusion:**

These results suggest that Nrf2 plays a crucial role in fibroblast activation and scar formation, and our findings offer a novel therapeutic approach for reducing scar formation after skin injury.

## 1 Introduction

Wound healing is a crucial process in maintaining skin homeostasis after injury, where extracellular matrix (ECM) remodeling plays a vital role. Any abnormalities in this process lead to excessive wound healing or scar formation. Currently, clinical measures such as surgery, laser therapy, and medication are widely used in scar treatment [1]. However, these methods often have significant drawbacks such as poor patient compliance and unsatisfactory efficacy [2]. Therefore, it is essential to further explore the pathogenesis of scar formation.

The scar is primarily composed of poorly structured and disordered collagen fibers, and the formation of the scar is closely related to ECM remodeling, with fibroblasts being the main cell type involved. In the process of wound healing, abnormally activated fibroblasts produce excessive amounts of collagen, hyaluronic acid, fibronectin, and proteoglycans [3]. This process leads to excessive collagen synthesis and the deposition of extracellular matrix by fibroblasts [4]. Although fibroblast activation is crucial in scar formation, the underlying mechanisms remain unclear and require further investigation. Recent studies have demonstrated that inflammatory factors and reactive oxygen species (ROS) play a crucial role in regulating fibroblast activation, a process that involves multiple signaling pathways [5]. Among these, the MAPK signaling pathway, particularly the ERK pathway, plays a crucial role in the activation and proliferation of fibroblasts. For example, ginsenoside Rg3 can inhibit scar fibroblast proliferation, angiogenesis, and collagen synthesis in vitro via the ERK signaling pathway [6], and BMP1 promotes inflammatory responses in scars through ERK pathway modulation [7]. Therefore, regulating fibroblast‐mediated inflammation and collagen synthesis via the ERK signaling pathway is critical for scar progression.

NF‐erythroid 2–related factor 2 (Nrf2) is a sensitive redox transcription factor. Nrf2 and its endogenous inhibitor, Keap1, play significant roles in regulating the balance of pro‐ and anti‐inflammatory responses and oxidative processes [8]. Studies have confirmed that Nrf2 is primarily involved in the proliferative phase of wound healing [9]. It accelerates wound healing by promoting epithelial cell proliferation and migration, while reducing oxidative stress and apoptosis [10]. Li Xue et al. found that in a diabetic rat model, exosomes secreted by adipose‐derived stem cells (ADSCs) overexpressing Nrf2 facilitate angiogenesis and wound healing of diabetic foot ulcers [11]. Ganesh et al. also discovered that Pterostilbene can accelerate wound healing by promoting Nrf2 expression [12]. These results suggest that Nrf2 plays a crucial role in wound‐healing process. Additionally, previous studies have demonstrated that Nrf2 deficiency promotes ERK pathway activation throughout the development and progression of dermatitis, resulting in a marked increase in inflammatory factors—such as IL‐1α, IL‐1β, and IL‐6—as well as ear thickening and swelling. These findings indicate the significant role of Nrf2 in mitigating skin inflammation. Considering that inflammation can trigger fibroblast activation, we speculate that Nrf2 may influence scar formation.

In this study, we used Nrf2 knockout (Nrf2‐KO) and Wild‐Type (WT) mice to explore the role of Nrf2 in scar formation. Our results revealed that Nrf2 deficiency may promote fibroblast activation through increasing inflammatory response and collagen deposition. Therefore, our findings highlight the significant role of Nrf2 in collagen synthesis and scar formation, providing new insights and potential molecular targets for scar treatment.

## 2 Materials and Methods

### 2.1 Experimental Animals

NF‐erythroid 2–related factor 2 (Nrf2)‐KO mice on a C57BL/6J background were obtained from Jackson Laboratory. Eight‐week‐old male mice were used in the current studies. Sex‐ and age‐matched C57BL/6J Wild‐Type mice were purchased from SiPeiFu Company (Beijing, China). Mice were randomly assigned to experimental groups using a randomization scheme to minimize selection bias. Full‐thickness punch biopsy wounds were made in Nrf2‐KO and Wild‐Type mice by using a 6‐mm punch on the dorsum of mice. Wound healing assessments were performed in a blinded manner during both the wound model construction and outcome measurement phases. The wound areas were measured using ImageJ software to evaluate the rate of wound healing until all wounds had completely healed on Day 12.

### 2.2 Hematoxylin and Eosin Staining

The scar tissue was embedded with paraffin and cut into 5 μm sections. Paraffin‐embedded sections were deparaffinized and stained with hematoxylin and eosin (HE). Images were taken with Nikon microscope.

### 2.3 Masson's Trichrome Staining

The deposition of collagen in scar was evaluated by using Masson's Trichrome Staining Kit (G1340, Solarbio).

### 2.4 Immunohistochemical (IHC) and Immunofluorescence (IF) Staining

The immunohistochemical staining was performed as described [13]. Skin sections were deparaffinized, and antigen retrieval was performed using sodium citrate antigen retrieval solution (C1032, Solarbio). Then, the sections were incubated in a 3% H2O2 methanol solution for 10 min, followed by incubation in 1% goat serum for 1 h to block non‐specific binding. The slides were sequentially incubated with rabbit anti‐mouse Vimentin antibody (2031‐1‐AP, Proteintech, 1:200), Proliferating Cell Nuclear Antigens (PCNA) antibody (CL488‐10205, Proteintech, 1:200) overnight at 4°C, and goat anti‐rabbit HRP (HS101‐01, TransGen Biotech, 1:200) for 1 h at room temperature, and the group of negative control was only incubated with goat anti‐rabbit HRP (HS101‐01, TransGen Biotech, 1:200) for 1 h at room temperature, followed by DAB reaction using DAB kit (ZLI‐9018, ZSBio).

To evaluate the nuclear localization of phosphorylated ERK, the cells were treated with 50 and 100 nM TPA (P‐8139; Sigma) for 30 min, then washed with PBS and fixed in 4% formaldehyde for 20 min. We permeated and blocked the cells using a 0.2% Triton X‐100 (T8200; Solarbio) solution and a 1% BSA solution (A8020; Solarbio). Subsequently, the cells were incubated with pERK (4370S, Cell Signaling Technology, 1:50) overnight at 4°C, and Multi‐rAb CoraLite Plus 488‐Goat Anti‐Rabbit Recombinant Secondary Antibody (H + L) (RGAR002, Proteintech, 1:1000) for 1 h at room temperature and the group of negative control was only incubated with Multi‐rAb CoraLite Plus 488‐Goat Anti‐Rabbit Recombinant Secondary Antibody (H + L) (RGAR002, Proteintech, 1:1000) for 1 h at room temperature and stained with DAPI (C0065; Solarbio).

### 2.5 Real‐Time Fluorescence Quantitative PCR

To evaluate the role of Nrf2 in scar formation, total RNA was isolated from the scar until it had healed completely on Day 12 using the TransZol Up reagent (ET111‐01, TransGen Biotech). cDNA was synthesized with All‐in‐One FirstStrand cDNA Synthesis SuperMix for RT‐PCR (AE341‐02, TransGen Biotech). Then qPCR was carried on the 7500‐Fast Real‐Time PCR System (Applied Biosystems, America), using PerfectStart Green qPCR SuperMix (AQ602‐24, TransGen Biotech). The relative mRNA levels of each sample were determined by the $2^{-∆∆C_{t}}$ method with the housekeeping gene glyceraldehyde‐3‐phosphate dehydrogenase (GAPDH). The primer sequences for qRT‐PCR are listed in Table 1.

**Table 1:** Primer sequences for qRT‐PCR.

Gene	Primer type	Sequnce (5′→ 3′)
Mus‐GAPDH	Forward (F)	AGGTCGGTGTGAACGGATTTG
	Reverse (R)	GGGGTCGTTGATGGCAACA
Mus‐α‐SMA	Forward (F)	TCGGATACTTCAGCGTCAGGA
	Reverse (R)	GTCCCAGACATCAGGGAGTAA
Mus‐COL1A1	Forward (F)	GGGTGGAGGGAGTTTACACG
	Reverse (R)	CAGTCGCTTCACCTACAGCA
Mus‐SOD1	Forward (F)	AACCAGTTGTGTTGTCAGGAC
	Reverse (R)	CCACCATGTTTCTTAGAGTGAGG
Mus‐SOD2	Forward (F)	CAGACCTGCCTTACGACTATGG
	Reverse (R)	CTCGGTGGCGTTGAGATTGTT
Mus‐HO‐1	Forward (F)	AAGCCGAGAATGCTGAGTTCA
	Reverse (R)	GCCGTGTAGATATGGTACAAGGA
Mus‐GSH1	Forward (F)	CTTCCCTCCCTTCGGATCG
	Reverse (R)	GTCCACAGAGATGCAGTGAAA
Mus‐COL3A1	Forward (F)	CCACCAGGACTGCCGTTATT
	Reverse (R)	TCACCAGGACAAAGAGGGGA
Mus‐COL6A1	Forward (F)	AACAGGAATAGGAAATGTGACCC
	Reverse (R)	ACACCACGGATAGGTTAGGGG
Mus‐COL17A1	Forward (F)	AAGTCACCGAGAGAATTGTCAC
	Reverse (R)	AGAGAGCCTGTCTTAGCATATCC
Mus‐Vimentin	Forward (F)	CGGCTGCGAGAGAAATTGC
	Reverse (R)	CCACTTTCCGTTCAAGGTCAAG
Mus‐FN1	Forward (F)	ATGTGGACCCCTCCTGATAGT
	Reverse (R)	GCCCAGTGATTTCAGCAAAGG
Mus‐MMP2	Forward (F)	CAAGTTCCCCGGCGATGTC
	Reverse (R)	TTCTGGTCAAGGTCACCTGTC
Mus‐MMP3	Forward (F)	CCTGGAACAGTCTTGGCTCATGC
	Reverse (R)	GGTACATCAGAGCTTCAGCCTTGG
Mus‐MMP8	Forward (F)	TCTTCCTCCACACACAGCTTG
	Reverse (R)	CTGCAACCATCGTGGCATTC
Mus‐MMP9	Forward (F)	TGATGTTATGATGGTCCCACTTG
	Reverse (R)	GCAGAGGCATACTTGTACCG
Mus‐IL‐1α	Forward (F)	CGAAGACTACAGTTCTGCCATT
	Reverse (R)	GACGTTTCAGAGGTTCTCAGAG
Mus‐IL‐1β	Forward (F)	TGGATGCTCTCATCAGGACAG
	Reverse (R)	GAAATGCCACCTTTTGACAGTG
Mus‐TGF‐β1	Forward (F)	CTGGCGAGCCTTAGTTTGGAC
	Reverse (R)	CCACCTGCAAGACCATCGAC
Mus‐IL‐6	Forward (F)	AGTGGTATAGACAGGTCTGTTGG
	Reverse (R)	CTGCAAGAGACTTCCATCCAG
Mus‐CXCL‐1	Forward (F)	TGGGGACACCTTTTAGCATCTT
	Reverse (R)	ACTGCACCCAAACCGAAGTC
Mus‐CXCL‐2	Forward (F)	GCGTCACACTCAAGCTCTG
	Reverse (R)	CCAACCACCAGGCTACAGG
Mus‐TIMP1	Forward (F)	CGAGACCACCTTATACCAGCG
	Reverse (R)	ATGACTGGGGTGTAGGCGTA
Mus‐TIMP2	Forward (F)	CTGGACGTTGGAGGAAAGAAG
	Reverse (R)	CTGGGTGATGCTAAGCGTGTC

### 2.6 Western Blot

Tissues on Day 12 were under homogenization with RIPA Lysis Buffer (CW2333S, CWbio) and 1 mM PMSF (P0100, Solarbio). Protein concentration was detected by using the Pierce TM BCA Protein Assay Kit (23227, Thermo). Protein bands were separated on 10% SDS‐PAGE gels by electrophoresis, and the Wild‐Type and Nrf2‐KO samples were on the same gel. Subsequently, the gels were transferred to 0.45 μm polyvinylidene fluoride membranes (IPVH00010, Immobilon). After blocking with 5% (w/v) non‐fat milk in TBST buffer (pH 7.5) for 1 h, the membranes were incubated with α‐SMA (ab32575, Abcam, 1:1000), COL6A1 (17023‐1‐AP, Proteintech, 1:1000), pERK (4370S, Cell Signaling Technology, 1:1000), ERK (9102S, Cell Signaling Technology, 1:1000), and β‐Actin polyclonal Ab (YT0099, Immunoway, 1:2000). Then membranes were incubated with ProteinFind Goat Anti‐Mouse IgG (H + L), HRP Conjugate (HS201‐01, TransGen Biotech, 1:4000) or ProteinFind Goat Anti‐Rabbit IgG (H + L), HRP Conjugate (HS101‐01, TransGen Biotech, 1:4000) for 1 h. Since the target protein weights (α‐SMA, ERK, pERK), the target protein antibodies were dissociated using Western Blot Fast Stripping Buffer (PS07S, Epizyme Biotech). Protein bands were visualized by a chemiluminescence imaging analysis system (Tanon 5200, China). The grayscale values of the blot were then quantified using ImageJ software.

### 2.7 Culture of Primary Skin Fibroblasts

Dermal fibroblasts were separated from the dorsal skin of newborn mice and cultured as previously described [13]. Cells were cultured in DMEM High Glucose (12416001, Corning) supplemented with 10% TransSerum EQ Fetal Bovine Serum (FS201‐02, TransGen Biotech) and 1% Penicillin‐Streptomycin (FG101‐01, TransGen Biotech) at 37°C with 5% CO_2_. After treatment with 50 and 100 nM TPA (P‐8139; Sigma) for 6 h, fibroblasts were harvested for the measurement of the expression of collagen synthesis‐related genes by real‐time fluorescence quantitative PCR.

### 2.8 Cell Migration Assay

The cells were cultured at a concentration of 1 × 10^6^ cells per well in a six‐well plate. Until the cells were 60%–80% confluent, a straight scratch was made using a P200 pipette tip. The cells were photographed every 3 h until the scratch wounds closed completely. The images were analyzed using ImageJ software to assess the speed of wound scratching.

### 2.9 Statistical Analysis

The unpaired Student's *t*‐test was performed to determine the significance values using GraphPad Prism 9.3.1. The data were expressed as means ± standard deviation (SD). A *p* value < 0.05 was accepted as statistically significant.

## 3 Results

### 3.1 Nrf2 Deficiency Delays Wound Healing and Exacerbates Scar Formation

To explore the role of Nrf2 in wound healing and scar formation, we measured the punch biopsies daily for 12 days (Figure 1A) and evaluated the area of wound healing (Figure 1B). We found that in the early stage of wound healing, Nrf2‐KO mice exhibited a significantly slower wound healing rate. We observed a significantly thicker epidermis in the scar area of Nrf2‐KO mice using HE staining (Figure 1C). To further investigate the collagen deposition in the scar, we found that Nrf2 deficiency significantly exacerbated the scar formation (Figure 1D). These results indicated that the absence of Nrf2 significantly delayed the early stage of healing and exacerbated scar formation.

**Figure 1:**
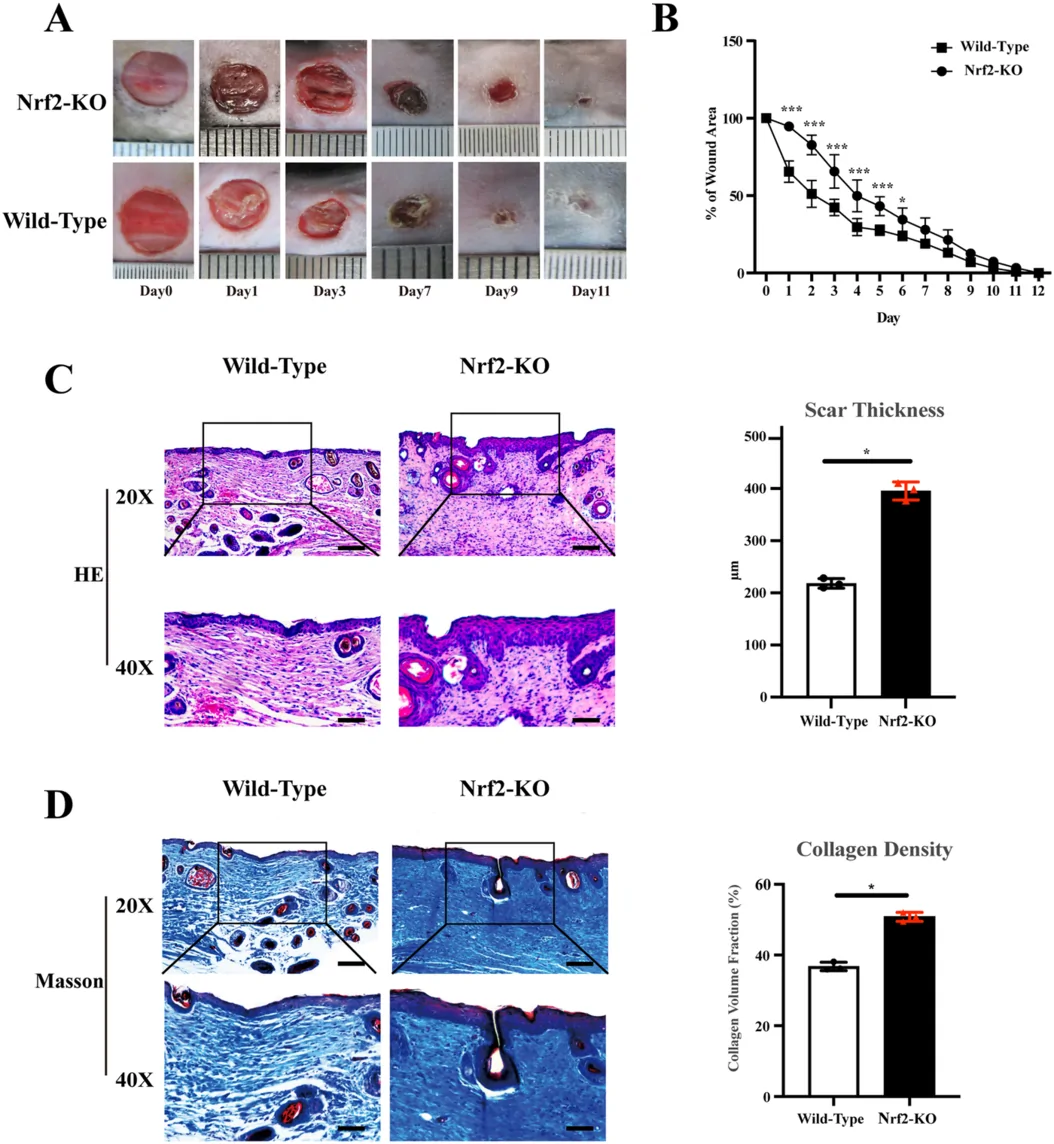
NF‐erythroid 2–related factor 2 (Nrf2) deficiency delays wound healing and exacerbates scar formation. Full‐thickness wounds were made on the dorsum of both the 8‐week‐old Nrf2‐KO and Wild‐Type mice to assess wound healing. (A) Representative macroscopic views of skin wounds. (B) The healing curves represent the ratio of the wound area to the initial area. The closure rate of injury was measured daily using a Vernier caliper and ImageJ software for 12 days. Each point represents the mean value for six mice per group. (C) Representative HE staining images. (D) Deposition of collagen was evaluated by Masson staining. Statistical significance was determined by two‐way ANOVA using Graphpad Prism 10.0. (*n* = 3 per group; Scale bars: 100 µm (20×), 50 µm (40×); *p < 0.05, ***p < 0.001).

### 3.2 The Deficiency of Nrf2 Exacerbates Collagen Synthesis of Scar In Vivo

Since the proliferation, migration and collagen synthesis of fibroblasts are essential for wound healing, we first assessed the expression of Proliferating Cell Nuclear Antigens (PCNA) and Vimentin (Figure 2A) by Immunohistochemical staining (IHC). No difference was observed between the two groups; however, recent studies have shown that inflammatory responses and oxidative stress play a crucial role in abnormal fibroblast activation [14]. Therefore, we evaluated the levels of inflammation and oxidative stress on Day 12 (Figure 2B). The results indicated that in Nrf2‐KO mice, the expression of inflammatory factors (IL‐6, CXCL‐1, CXCL‐2, TGF‐β1) were elevated, while anti‐oxidative stress factors (SOD1, SOD2, HO‐1, GSH1) were attenuated. These suggested that Nrf2 may influence scar formation by modulating inflammation and oxidative stress levels.

**Figure 2:**
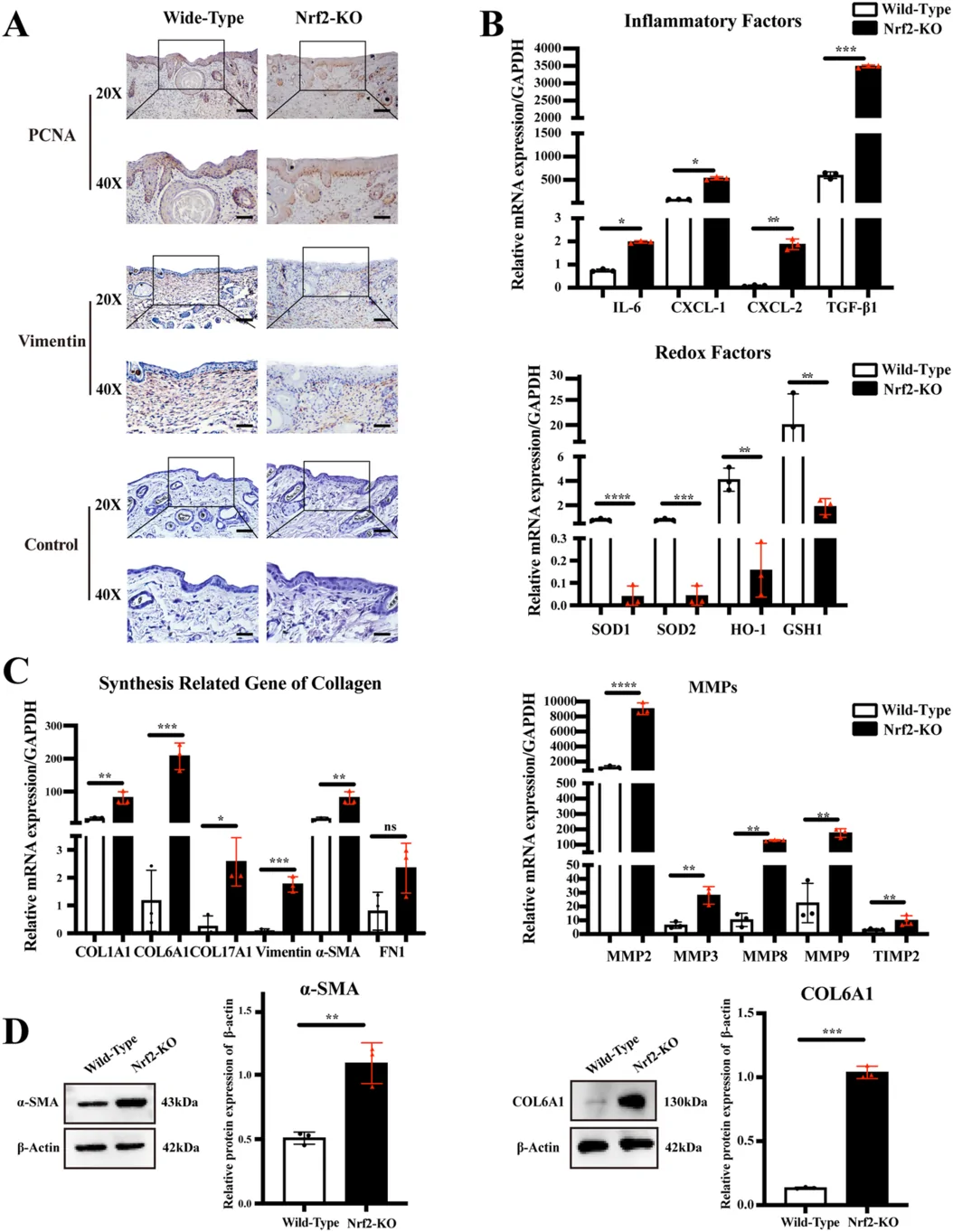
The deficiency of Nrf2 promotes collagen synthesis in the scar in vivo. (A) The expressions of Proliferating Cell Nuclear Antigens (PCNA) and Vimentin from different groups were verified by Immunohistochemical staining (IHC). In the negative control group, only the HRP secondary antibody was added to exclude the influence of non‐specific binding. (B) qRT‐PCR was used to evaluate the expression of inflammatory factors and redox factors on Dy 12. (C) qRT‐PCR was used to detect the expression of collagen synthesis‐related genes on day 12. (D) The protein levels of α‐smooth muscle actin (α‐SMA, 43 kDa) and Collagen VI alpha 1 chain (COL6A1, 130 kDa) were detected by Western blot on Day 12. (*n* = 3 per group; Scale bars: 100 µm (20×), 50 µm (40×), *p < 0.05, **p < 0.01, ***p < 0.001, ****p < 0.0001).

Meanwhile, in the scar formation, the dynamic balance between collagen synthesis and degradation is primarily mediated by matrix metalloproteinases (MMPs) [15]. In order to investigate collagen synthesis in the scar further, we used qRT‐PCR to detect the expression levels of collagen synthesis‐related factors (COL1A, COL6A1, COL17A1, Vimentin, α‐SMA, FN1) and MMPs (MMP2, MMP3, MMP8, MMP9, TIMP2) (Figure 2C). The results showed that the expression levels of these genes were significantly upregulated in Nrf2‐KO mice. Additionally, the protein expression levels of α‐SMA and COL6A1 in the scar tissue were also higher in Nrf2‐KO mice (Figure 2D). These findings indicated that Nrf2 deficiency promoted collagen synthesis and deposition in the scar.

### 3.3 Deficiency of Nrf2 Exacerbates Collagen Synthesis of Fibroblasts In Vitro

Fibroblast is a determinant of wound healing and is involved in such processes as ECM remodeling and collagen structures [16]. To further validate the results in vivo, we found that Nrf2‐KO inhibited fibroblasts proliferation and migration (Figure 3A,B). Subsequently, we stimulated fibroblasts with 50 and 100 nM TPA. Then we detected collagen expression levels 6 h after stimulation (Figure 3C). The results showed that the expression levels of inflammatory and collagen‐related factors in the fibroblasts were upregulated after TPA stimulation, and Nrf2 deficiency significantly promoted the expression of these genes compared with Wild‐Type fibroblasts. These findings suggested that Nrf2‐KO inhibited cell migration and exacerbated collagen synthesis of fibroblasts in vitro.

**Figure 3:**
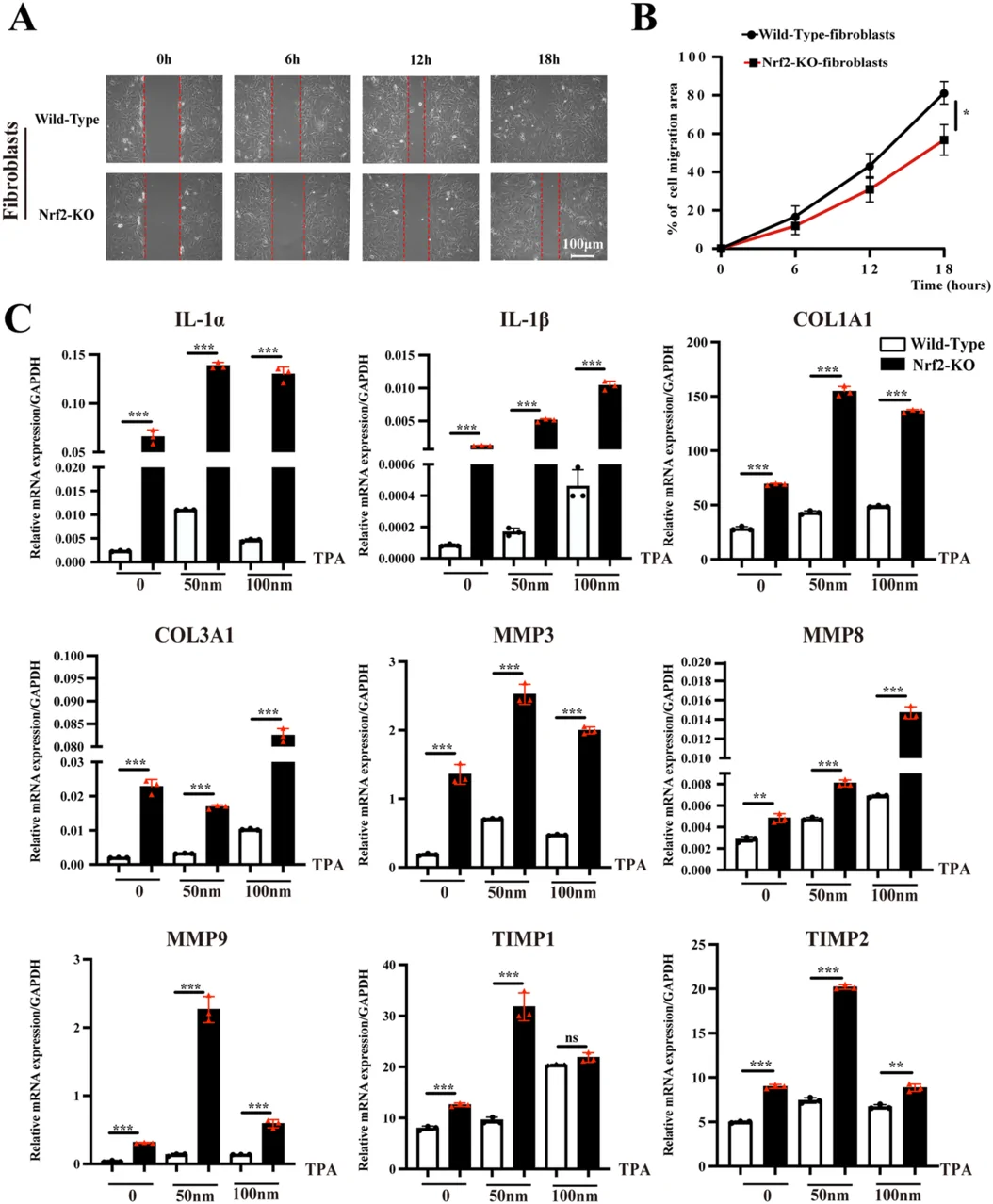
Deficiency of Nrf2 exacerbates migration and collagen synthesis of fibroblasts in vitro. (A) The cells were cultured at a concentration of 1 × 10^6^ cells per well in a six‐well plate. Until the cells were 60%–80% confluent, straight scratch was made with a p200 pipette tip. (B) The front velocity of the cells that migrated into each scratch area was analyzed by Image J. (C) The fibroblasts of Nrf2‐KO and Wild‐Type were treated with 50 and 100 nM Phorbol 12‐myristate 13‐acetate (TPA) for 6 h. Then qRT‐PCR was used to detect the expression of collagen synthesis‐related genes. (*n* = 3 per group; Scale Bar = 100 μm; *p < 0.05, **p < 0.01, ***p < 0.001).

### 3.4 Nrf2 Deficiency Exacerbates Scar Through Signaling Pathway In Vivo and In Vitro

Scar formation is closely related to fibroblast activation. Our previous study found that after TPA stimulation in vitro, the phosphorylated level of ERK in fibroblasts of Nrf2‐KO was elevated [17]. The results indicated that Nrf2 deficiency may activate fibroblasts through MAPK‐signaling pathway. Therefore, we detected the pERK level in vivo. The results showed that Nrf2 deficiency significantly upregulated ERK phosphorylation in vivo (Figure 4A). Meanwhile, after TPA stimulation, we detected the nuclear localization of phosphorylated ERK in fibroblasts by immunofluorescence staining. The results revealed that Nrf2‐KO significantly promoted nuclear localization of pERK (Figure 4B). Overall, these findings implied that Nrf2 deficiency may exacerbate scar through ERK‐signaling pathway (Figure 4C).

**Figure 4:**
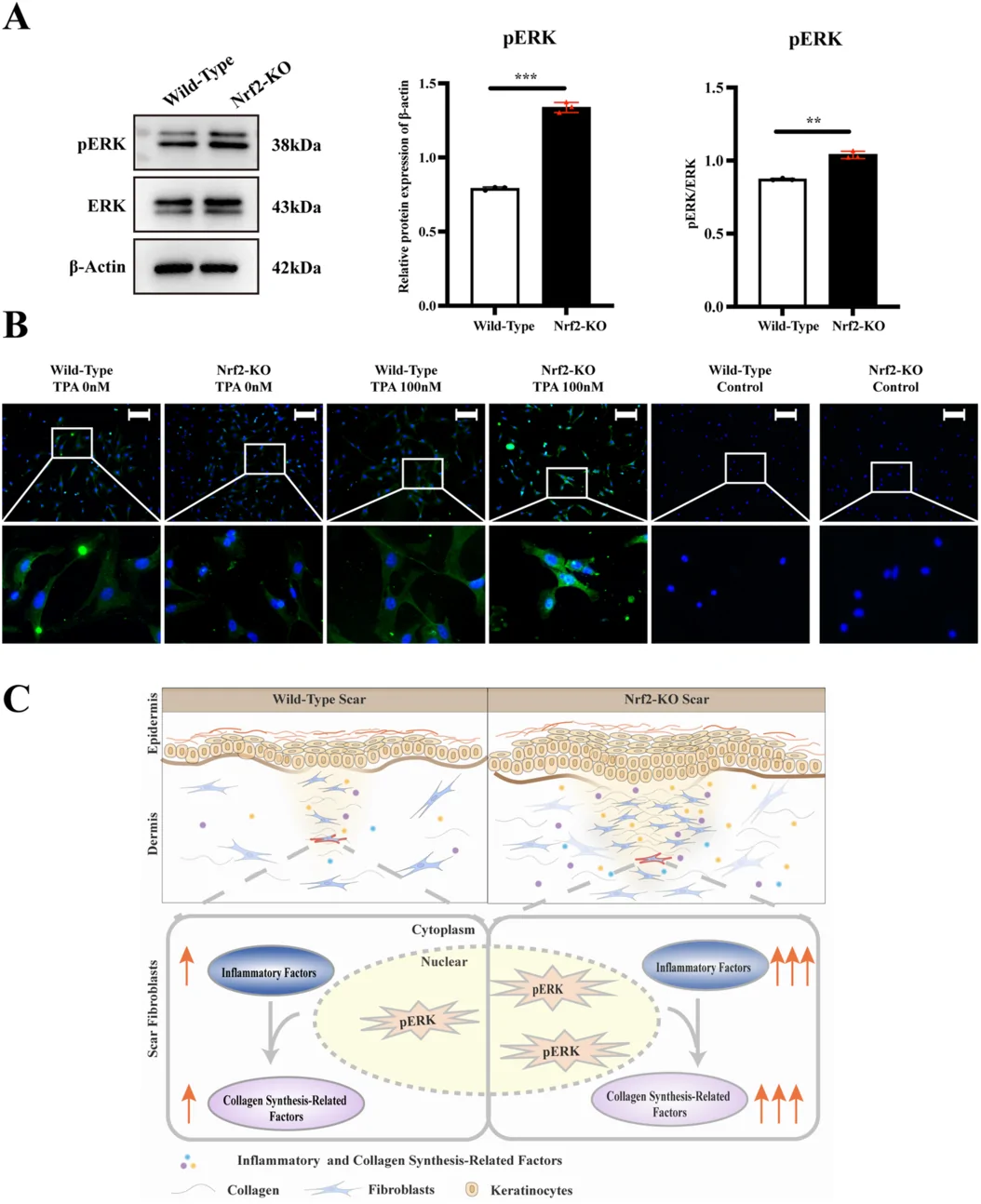
Nrf2 deficiency exacerbates scar through ERK signaling pathway in vivo and vitro. (A) The phosphorylated level of ERK (pERK, 38 kDa) in the scar was evaluated by Western Blot on Day 12. (B) Immunofluorescence staining was used to detect the nuclear localization of pERK from different groups. (C) The brief mechanism diagram of this study. (*n* = 3 per group; Scale Bar = 100 μm; **p < 0.01, ***p < 0.001).

## 4 Discussion

Inflammation is a crucial phase in wound healing. Severe or prolonged inflammatory responses are directly linked to delayed wound healing and scar formation [18]. Studies have shown that Nrf2 plays a significant role in the inflammatory response [19]. Our previous studies found that after TPA stimulation of Nrf2‐KO and Wild‐Type fibroblasts, Nrf2 deficiency upregulated many inflammatory factors such as IL‐1α and IL‐6 in fibroblasts, which are closely related to collagen synthesis. Recent research showed that IL‐6 can reduce the production of fibronectin and promote collagen deposition in keloid; IL‐1α is structurally related to fibroblast growth factors and is involved in skin repair [20, 21]. These results suggest that the absence of Nrf2 may affect collagen synthesis by promoting inflammatory response. In addition, the expression of collagen synthesis‐related genes plays a crucial role in scar formation. Paul Hiebert et al. found that Nrf2 can inhibit collagen mRNA expression in fibroblasts [22]. Our study also found that the absence of Nrf2 resulted in higher expression of collagen synthesis‐related genes such as MMP3, MMP8, MMP9, COL1A1, and COL3A1 in fibroblasts. This indicates that Nrf2 deficiency may directly regulate the expression of collagen synthesis‐related genes. Moreover, after the treatment of TPA, the increased expression of collagen synthesis‐related genes becomes more pronounced compared with the control group. This suggests that Nrf2 deficiency may exacerbate scar formation by activating inflammation, promoting fibroblast activation, and consequently enhancing collagen synthesis in fibroblasts. Overall, on the one hand, Nrf2 can directly regulate the expression of collagen‐related synthesis genes. On the other hand, it may modulate inflammatory response to regulate the expression of collagen synthesis‐related genes, thereby influencing collagen synthesis and scar formation.

Recent studies have found that scar formation is related to signaling pathways, including MAPK, TGF‐β/Smad, Integrins/FAK, Wnt/β‐catenin, and PI3K/Akt [23, 24, 25]. Wang Weidong et al. revealed that MAPK signaling pathway plays an important role in the formation of proliferative scars [26]. In our previous studies, the phosphorylated level of ERK in Nrf2‐KO was upregulated after TPA stimulation in vitro. Additionally, after using two ERK inhibitors, U0126 and PD98059, the pERK level was significantly decreased in Nrf2‐KO fibroblasts [17], indicating that Nrf2 deficiency may activate fibroblasts and promote inflammatory responses through the ERK‐signaling pathway. Many studies also indicate that ERK activation is closely associated not only with inflammation but also with fibroblast‐mediated collagen deposition. For instance, autophagy activates the MAPK/ERK pathway in renal tubular cells, inducing EGR1‐mediated upregulation of FGF2, which is secreted to stimulate fibroblasts and promote collagen synthesis and fibrosis [27]; fibroblast GSK‐3α promotes myofibroblast transformation and collagen synthesis via the ERK pathway, driving cardiac fibrosis independently of the canonical TGF‐β1‐SMAD3 pathway [28]; and ERK signaling mediates collagen synthesis and fibrotic activity in keloid fibroblasts, where SIRT6 overexpression inhibits ERK activity and thereby reduces fibroblast proliferation, migration, and collagen production [29]. Consistently, our data show that TPA stimulation increases collagen expression in Nrf2‐KO MEFs compared with WT cells (Figures 2 and 3), while treatment with ERK inhibitors U0126 and PD98059 suppresses collagen deposition (Supporting Information S1: Figure S1). Taken together, these findings suggest that Nrf2 may inhibit fibroblast‐mediated inflammation through the ERK‐signaling pathway, thereby suppressing abnormal collagen synthesis and slowing wound healing. In addition, the MAPK‐signaling pathway also includes other branches such as p38 and JNK, and we plan to further investigate the regulatory effects of NRF2 on these pathways in future studies.

Additionally, Nrf2 is a key redox transcription factor and regulates oxidative stress response [30, 31]. Meanwhile, oxidative stress also plays a crucial role in scar formation. Barrow RE et al. found that IL‐1 can promote oxidative stress in fibroblasts, affect collagen deposition, and enhance the formation of proliferative scars [32]. These suggest that Nrf2 is closely related to scar formation and oxidative stress. Our previous studies also found that Nrf2 deficiency enhanced inflammatory response through downregulating the expression of antioxidant factors such as HO‐1 in fibroblasts. Therefore, we detected the levels of oxidative stress factors in the scar, and the results were consistent with those of previous studies. This indicates that Nrf2 may also influence scar formation through oxidative stress pathways.

Although this study has yielded some important findings, several limitations remain. First, our research was primarily based on mouse models and fibroblast cultures, which may not fully reflect the complex process of scar formation in humans. Second, the mechanistic exploration in this study primarily focused on inflammation, oxidative stress, and the ERK branch of the MAPK‐signaling pathway, while other MAPK pathways, such as p38 and JNK, remain to be further investigated. Third, we primarily focused on fibroblasts, whereas keratinocytes, immune cells, and endothelial cells also play important roles in scar formation, but were not fully addressed in this study. Finally, this study lacks validation in human clinical scar samples, and therefore, its translational potential remains to be further confirmed.

Future studies should integrate multiple signaling pathways to more comprehensively elucidate the role of Nrf2 in scar formation. The use of advanced techniques, such as single‐cell sequencing, may help reveal the interactions between fibroblasts and other resident skin cells. In addition, validating these findings in human scar tissues and exploring the feasibility of modulating Nrf2 through pharmacological or genetic approaches may provide new directions for scar therapy. Long‐term studies will also be valuable to assess whether targeting Nrf2 can not only improve scar formation but also promote functional recovery after skin injury.

## Author Contributions

**Xueyuan Hao, Mengke Sun, Ruotong Wang, and Hong Chen:** writing – original draft, methodology, investigation, data curation. **Jing Luo and Junkai Huang:** formal analysis, software, methodology, investigation. **Haoyue Yu, Weibin Xing, and Yingxi Li:** supervision, validation, investigation. **Xiaoxia Li and Lizhi Hu:** conceptualization, resources, writing – review and editing. All authors approved the final version of the manuscript.

## Consent

The authors have nothing to report.

## Conflicts of Interest

The authors declare no conflicts of interest.

## Supporting information


**Figure S1:** TPA‐induced inflammation and collagen synthesis are attenuated using MAPK inhibitors.

Supporting Information

## Data Availability

All data generated or analyzed during this study are included in this published article [and its supplementary information files].
