# Supplementary material for: Deficiency of NF‐Erythroid 2‐Related Factor 2 Exacerbates the Scar Through ERK Signaling Pathway
Source: Immun Inflamm Dis. 2026 Feb 23;14(2):e70361. doi: 10.1002/iid3.70361 (PMC13757331; doi:10.1002/iid3.70361)
Supplement: Supplementary file 1 [file IID3-14-e70361-s001.pdf]

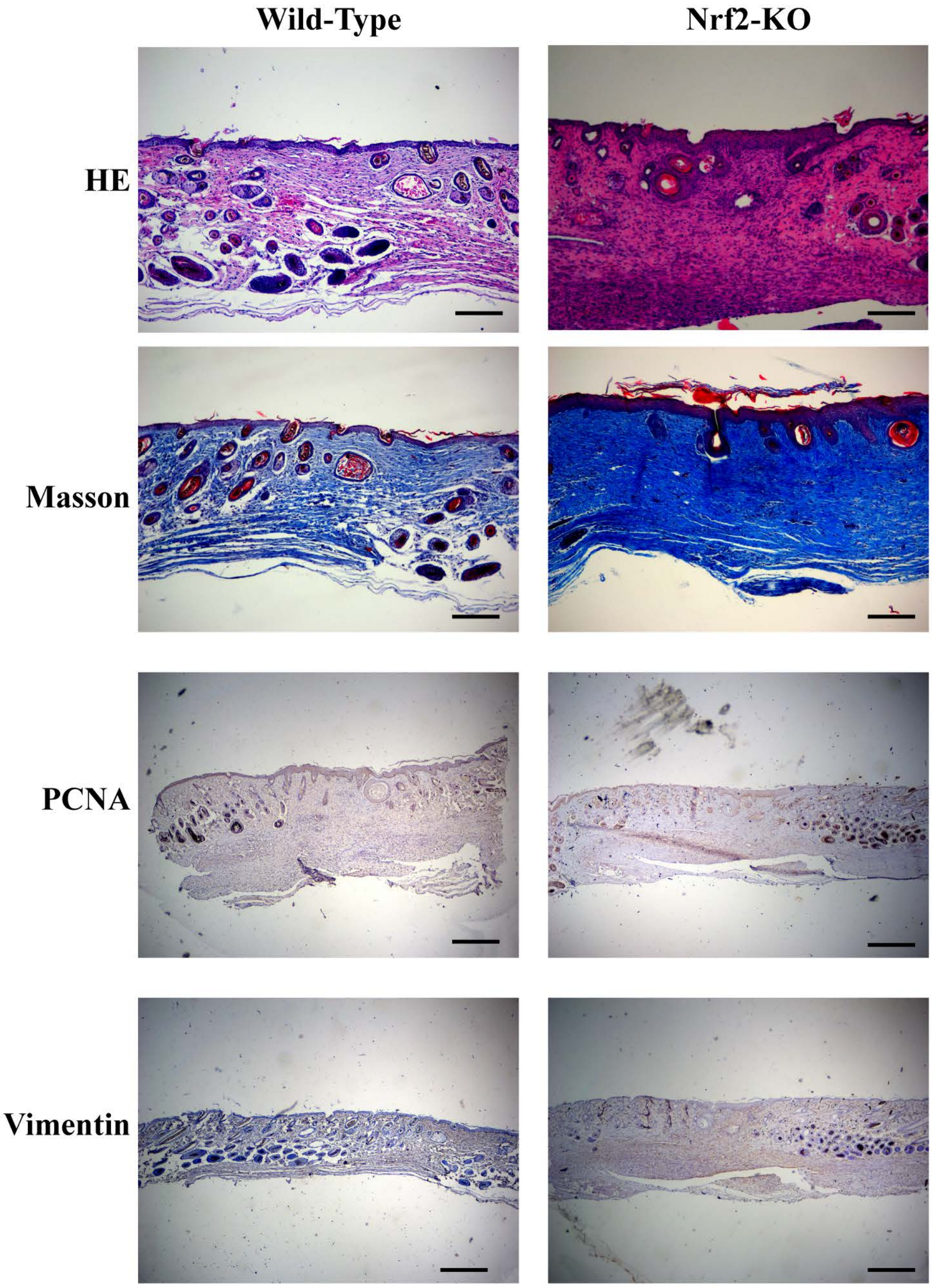
Wild-Type
Nrf2-KO
HE
Masson
PCNA
Vimentin

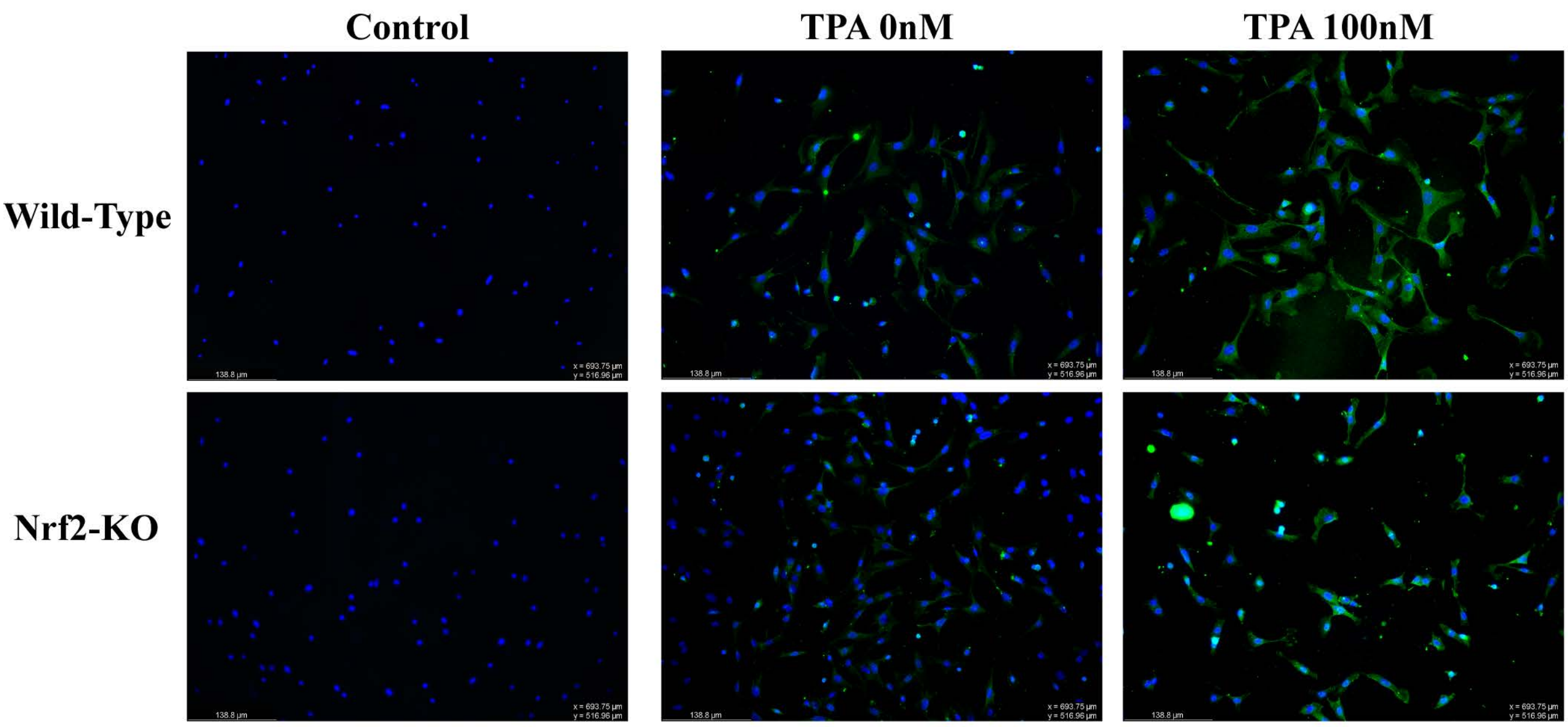
Control
TPA 0nM
TPA 100nM
Wild-Type
Nrf2-KO
