# Supplementary material for: Deficiency of NF‐Erythroid 2‐Related Factor 2 Exacerbates the Scar Through ERK Signaling Pathway
Source: Immun Inflamm Dis. 2026 Feb 23;14(2):e70361. doi: 10.1002/iid3.70361 (PMC13757331; doi:10.1002/iid3.70361)
Supplement: Supplementary file 2 [file IID3-14-e70361-s002.pdf]

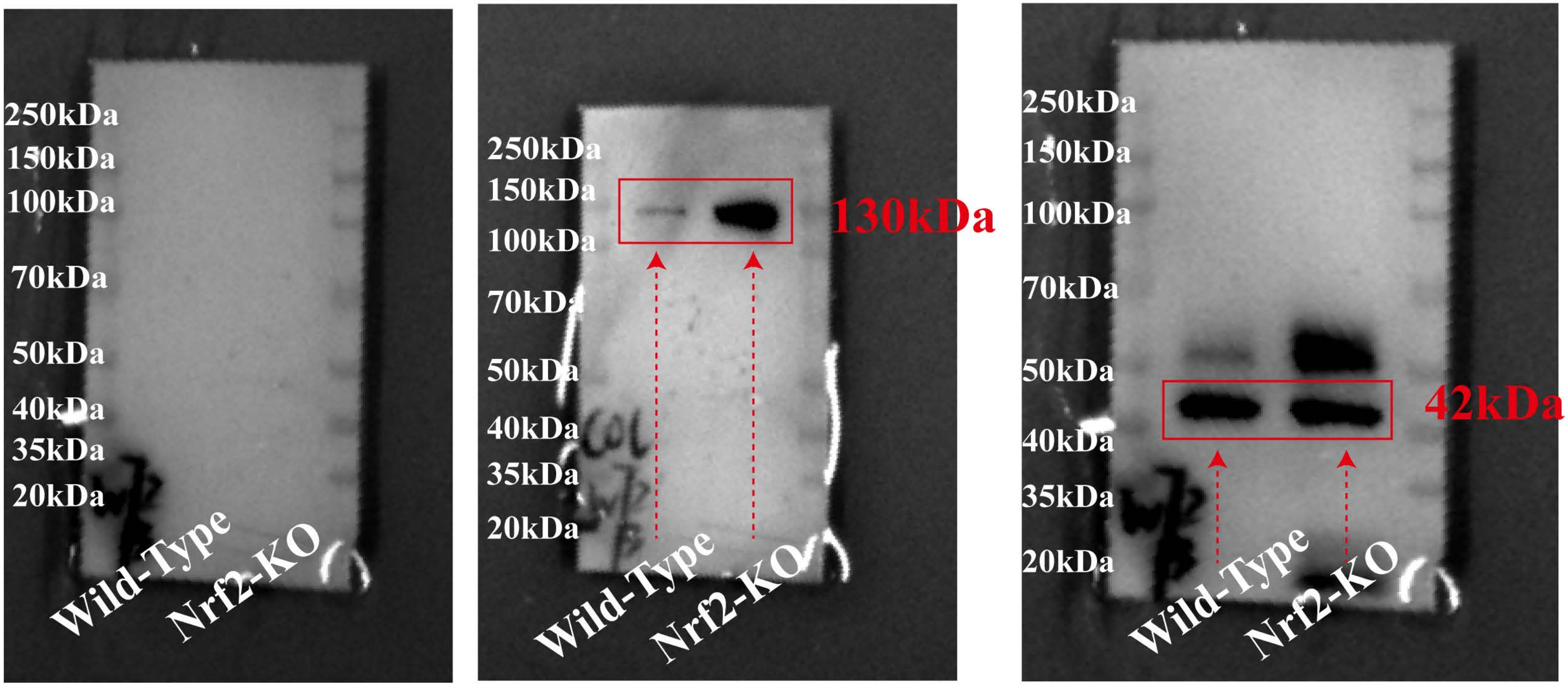

COL6A1+β-Actin   COL6A1   β-Actin

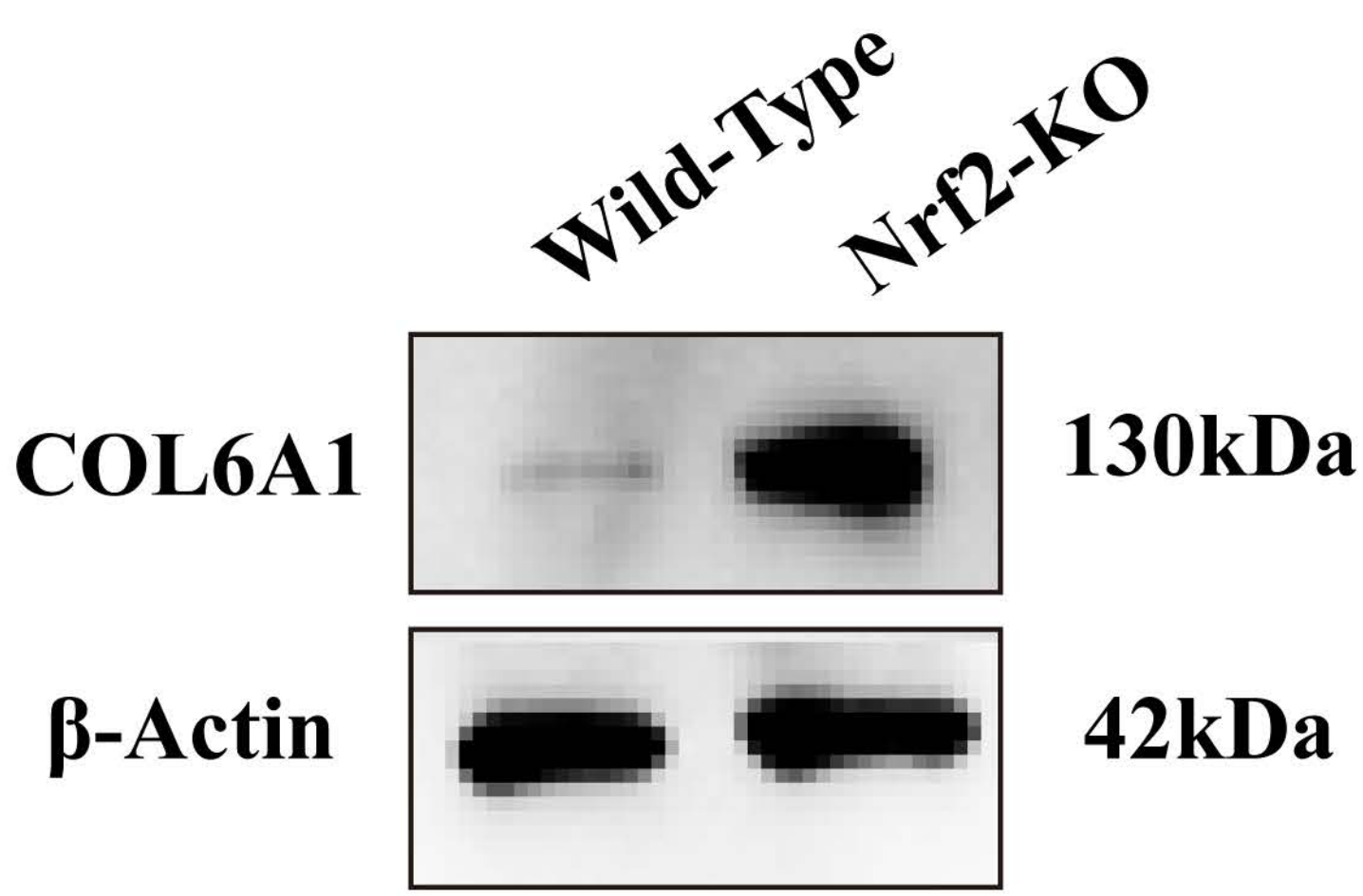

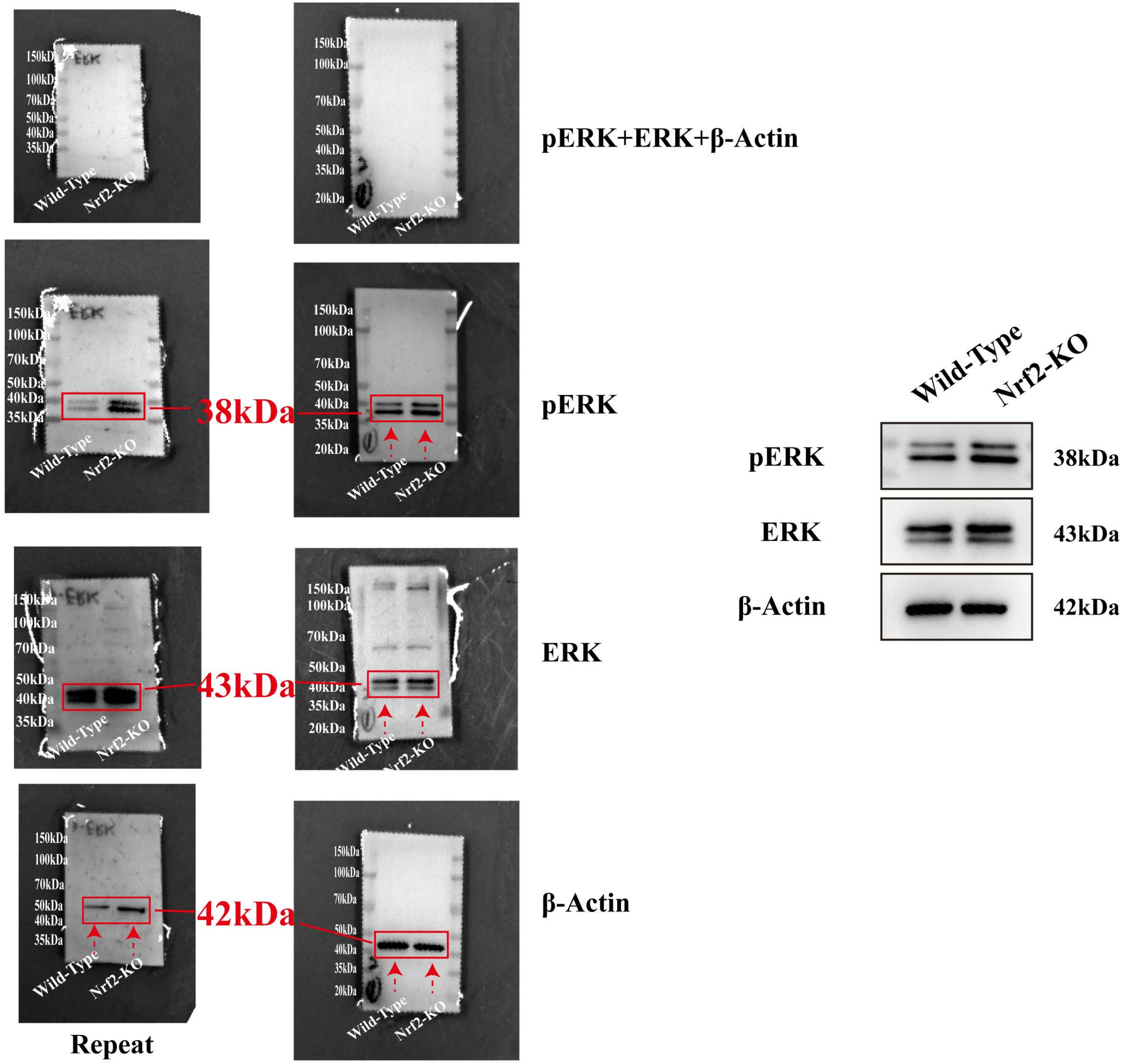

pERK+ERK+β-Actin
pERK
ERK
β-Actin
38kDa
43kDa
42kDa
Wild-Type
Nrf2-KO
150kDa
100kDa
70kDa
50kDa
40kDa
35kDa
20kDa
Repeat

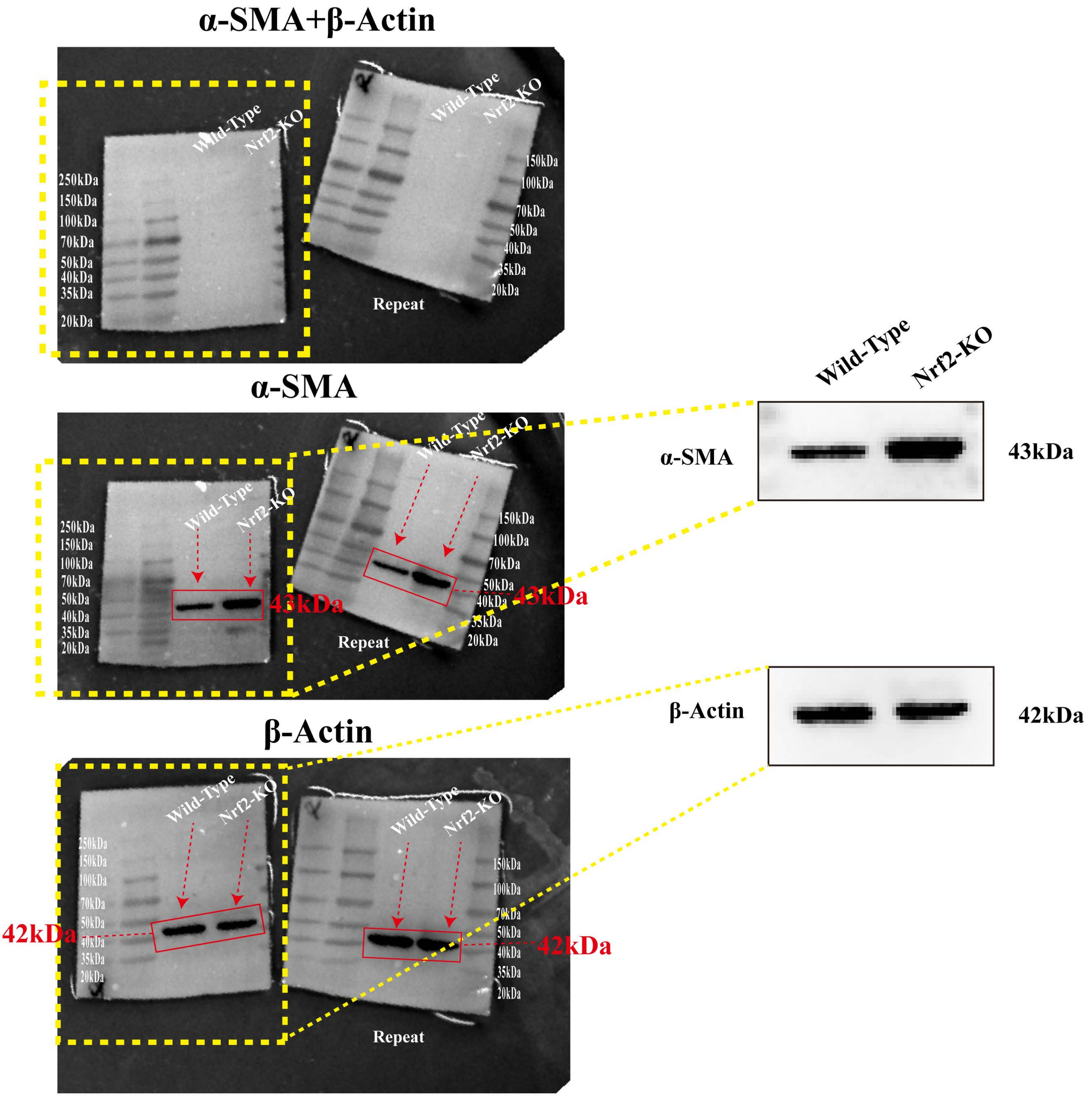

α-SMA+β-Actin
Wild-Type
Nrf2-KO
250kDa
150kDa
100kDa
70kDa
50kDa
40kDa
35kDa
20kDa
Repeat
α-SMA
43kDa
β-Actin
42kDa
